# Population-Level Interest and Telehealth Capacity of US Hospitals in Response to COVID-19: Cross-Sectional Analysis of Google Search and National Hospital Survey Data

**DOI:** 10.2196/18961

**Published:** 2020-04-07

**Authors:** Young-Rock Hong, John Lawrence, Dunc Williams Jr, Arch Mainous III

**Affiliations:** 1 Department of Health Services Research, Management and Policy College of Public Health and Health Professions University of Florida Gainesville, FL United States; 2 Biomedical Sciences Graduate Program College of Medicine The Ohio State University Columbus, OH United States; 3 Center for the Advancement of Team Science, Analysis, and Systems Thinking in Health Services and Implementation Science Research College of Medicine The Ohio State University Columbus, OH United States; 4 Department of Health Care Leadership and Management College of Health Professions Medical University of South Carolina Charleston, SC United States; 5 Department of Community Health and Family Medicine College of Medicine University of Florida Gainesville, FL United States

**Keywords:** COVID-19, telehealth, telemedicine, screening, pandemic, outbreak, infectious disease, public health

## Abstract

**Background:**

As the novel coronavirus disease (COVID-19) is widely spreading across the United States, there is a concern about the overloading of the nation’s health care capacity. The expansion of telehealth services is expected to deliver timely care for the initial screening of symptomatic patients while minimizing exposure in health care facilities, to protect health care providers and other patients. However, it is currently unknown whether US hospitals have the telehealth capacity to meet the increasing demand and needs of patients during this pandemic.

**Objective:**

We investigated the population-level internet search volume for telehealth (as a proxy of population interest and demand) with the number of new COVID-19 cases and the proportion of hospitals that adopted a telehealth system in all US states.

**Methods:**

We used internet search volume data from Google Trends to measure population-level interest in telehealth and telemedicine between January 21, 2020 (when the first COVID-19 case was reported), and March 18, 2020. Data on COVID-19 cases in the United States were obtained from the Johns Hopkins Coronavirus Resources Center. We also used data from the 2018 American Hospital Association Annual Survey to estimate the proportion of hospitals that adopted telehealth (including telemedicine and electronic visits) and those with the capability of telemedicine intensive care unit (tele-ICU). Pearson correlation was used to examine the relations of population search volume for telehealth and telemedicine (composite score) with the cumulative numbers of COVID-19 cases in the United States during the study period and the proportion of hospitals with telehealth and tele-ICU capabilities.

**Results:**

We found that US population–level interest in telehealth increased as the number of COVID-19 cases increased, with a strong correlation (*r*=0.948, *P*<.001). We observed a higher population-level interest in telehealth in the Northeast and West census region, whereas the proportion of hospitals that adopted telehealth was higher in the Midwest region. There was no significant association between population interest and the proportion of hospitals that adopted telehealth (*r*=0.055, *P*=.70) nor hospitals having tele-ICU capability (*r*=–0.073, *P*=.61).

**Conclusions:**

As the number of COVID-19 cases increases, so does the US population’s interest in telehealth. However, the level of population interest did not correlate with the proportion of hospitals providing telehealth services in the United States, suggesting that increased population demand may not be met with the current telehealth capacity. Telecommunication infrastructures in US hospitals may lack the capability to address the ongoing health care needs of patients with other health conditions. More practical investment is needed to deploy the telehealth system rapidly against the impending patient surge.

## Introduction

As the novel coronavirus diseases (COVID-19) spreads widely across the United States, telehealth capabilities have never been more important [1]. To boost telehealth use in response to COVID-19, the Centers for Medicare and Medicaid Services (CMS) has now expanded telehealth services for all Medicare beneficiaries [2], and national health agencies have urged health care providers to implement telehealth systems [3]. Virtually all electronic communications between patients and providers, including asynchronous modalities (eg, virtual check-ups or electronic visits [e-visits]) and real-time communication (eg, videoconferencing) can now be paid at the same rate as in-person visits [2].

This expansion of telehealth services is expected to alleviate the overload of the nation’s health care capacity by delivering timely care for initial screening of symptomatic patients (eg, forward triage) and potentially keep them away from hospitals to protect clinicians and other patients [2-5]. Although a massive surge of patients with COVID-19 or other pre-existing conditions is projected [6], it is currently unknown whether US hospitals have the telehealth capacity to meet the increasing demand and needs of patients. To address this gap and provide a snapshot of telehealth capacity in the United States, we investigated the relationship of population-level internet search volume for telehealth (as a proxy of population interest and demand) with the number of new COVID-19 cases and the proportion of hospitals that adopted the telehealth system (eg, telehealth capacity) in US states. Because a large concern with COVID-19 cases is the potential need for ICU beds and ventilators, we also identified the telemedicine intensive care unit (tele-ICU) capacity of US hospitals. Tele-ICU is “technology-enabled care delivered from off-site locations that was developed to address the increasing complexity of patients and insufficient supply of intensivists” [7]. As COVID-19 cases in the United States increase exponentially, tele-ICU may be able to provide an additional layer of care remotely, potentially easing some of the expected forthcoming capacity constraints.

## Methods

We used internet search volume data from Google Trends [8]. Given that Google Search is the most widely used search engine, we assumed users’ search volume would represent a national interest in telehealth [9]. We used two search terms—“telehealth” and “telemedicine”—since they are frequently used interchangeably. To compare the population interest with the trends in COVID-19, we obtained search data from January 21, 2020 (when the first COVID-19 case reported), and March 18, 2020 (most current data available). Search data are presented using a relative search volume (RSV) index ranging from 0 to 100, where 100 indicates the peak of search volume. For example, if the RSV is 70, 70% of the highest search volume is recorded, given the search period, geographic area, and population size [7]. Data on COVID-19 cases in the United States were obtained from the Johns Hopkins Coronavirus Resources Center [10]. To determine whether a hospital provides telehealth services (including telemedicine, e-visit, remote monitoring), we obtained data from the 2018 American Hospital Association Annual Survey (AHAAS) and the AHAAS Information Technology (IT) Supplement [11]. We estimated the proportion of hospitals that adopted the telehealth system and tele-ICU capacity by combining positive responses to the AHAAS survey and the IT supplement questions. We used Pearson correlation to examine the association of the population search volume for telehealth and telemedicine (composite RSV score) with the cumulative numbers of COVID-19 cases in the United States during the study period and the proportion of hospital-level telehealth and tele-ICU capabilities. The level of search volume and telehealth capability by quintiles were also mapped using state-level Federal Information Processing Standards codes. All analysis was conducted using SPSS version 26 (IBM Corporation) and SAS version 9.4 (SAS Institute Inc).

## Results

**Figure 1:**
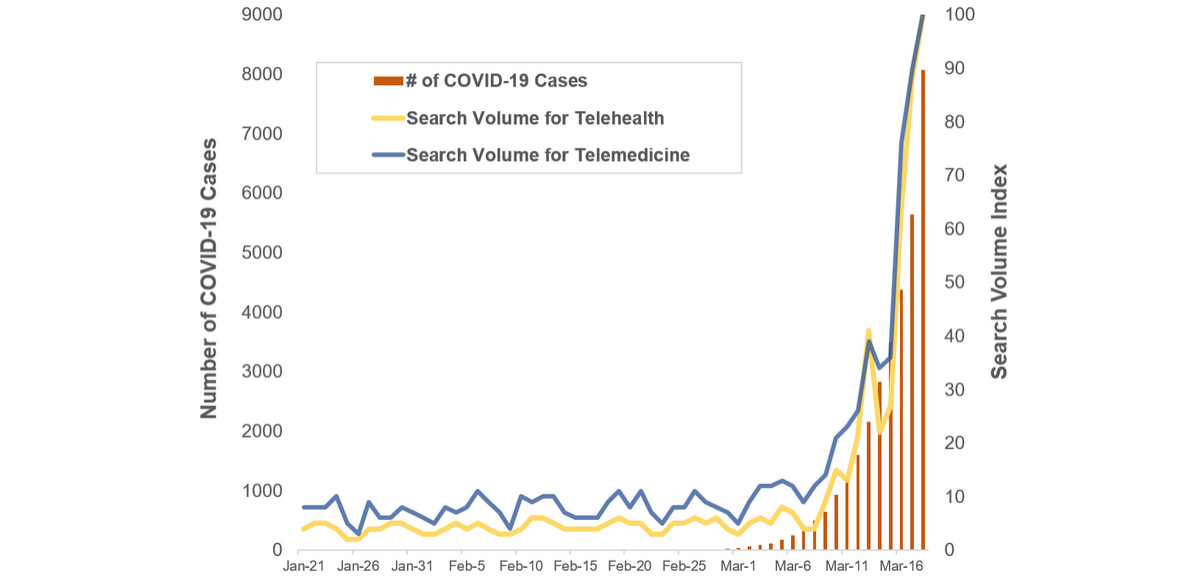
Trends in search volume for telehealth and the number of COVID-19 cases in the United States.

**Figure 2:**
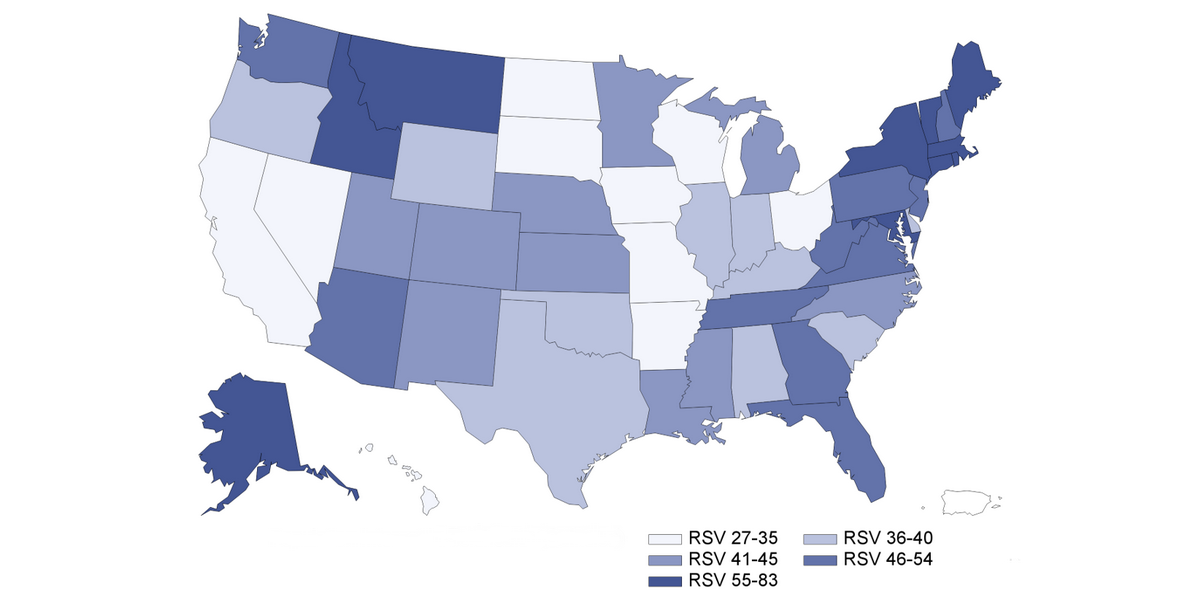
Population interest in telehealth by US state. RSV: relative search volume.

**Figure 3:**
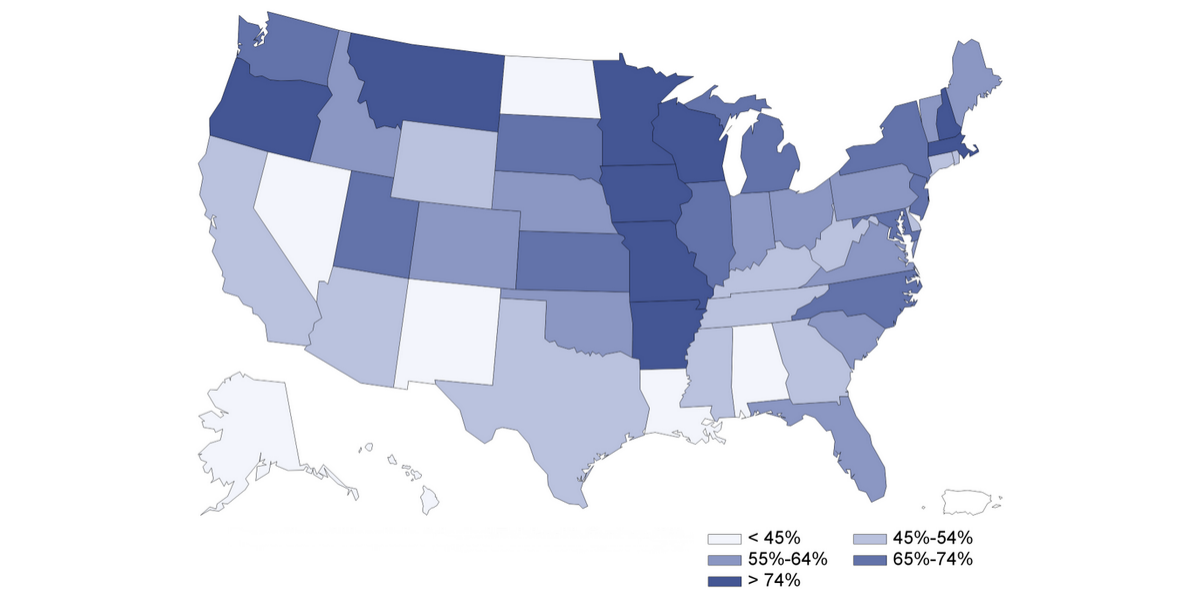
Proportion of hospitals that adopted the telehealth system by US state. RSV: relative search volume.

**Figure 4:**
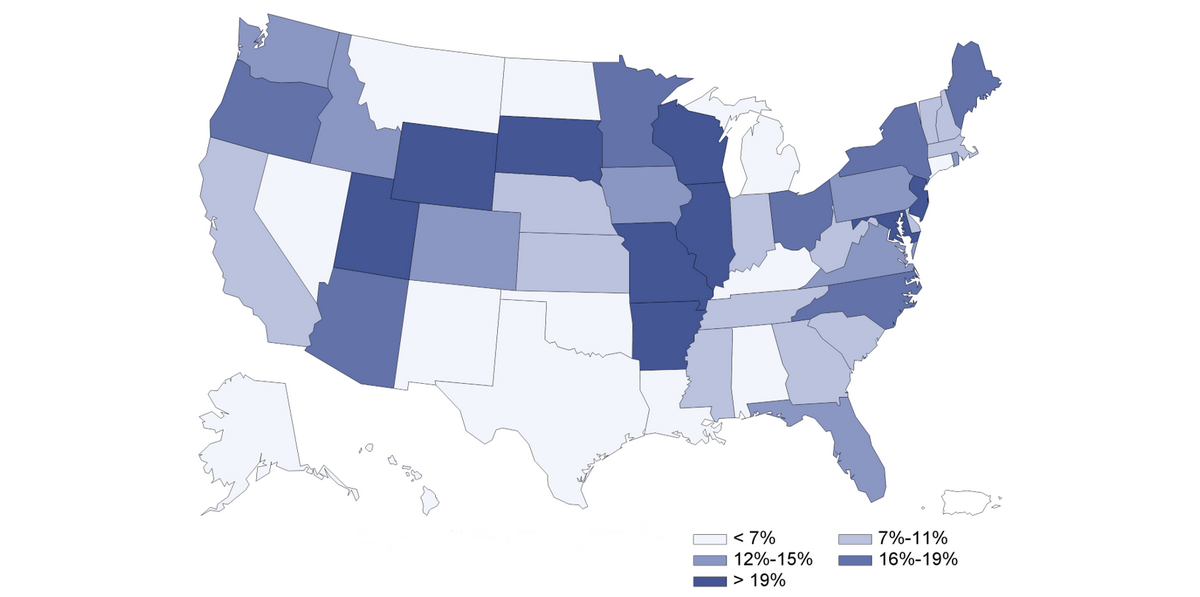
Proportion of hospitals having telemedicine intensive care unit capability by US state. RSV: relative search volume; ICU: intensive care unit.

The US population’s interest in telehealth increased as the number of COVID-19 cases increased (Figure 1). There was a strong correlation between population interest and COVID-19 cases reported (*r*=0.948, *P*<.001). Figure 2 presents the state-level population interest in telehealth (5 quintiles). Figures 3 and 4 show the proportion of hospitals with telehealth and tele-ICU capabilities. Of the 6146 US hospitals included, 3727 (60.8%) adopted telehealth and 788 (13.4%) had tele-ICU capability. We observed a higher population interest in telehealth in the Northeast and West census region (Figure 3), whereas the proportion of hospitals that adopted telehealth was higher in Midwest region (Figure 4). There was no significant association between population interest and proportion of hospitals that adopted telehealth (*r*=0.055, *P*=.70) nor hospitals having tele-ICU capability (*r*=–0.073, *P*=.61). The proportion of hospitals with telehealth and tele-ICU capabilities in the 50 states are listed in Table 1.

**Table 1:** Proportion of hospitals having telehealth and telemedicine intensive care unit capabilities in the 50 US states^a^.

States	Telehealth system (%)	Tele-ICU^b^ (%)
Alabama	39.7	0.9
Alaska	40.7	7.4
Arizona	50.9	17.9
Arkansas	79.4	22.5
California	51.3	7.8
Colorado	62.5	13.5
Connecticut	53.5	2.3
Delaware	46.2	7.7
Florida	59.1	12.8
Georgia	54.3	7.5
Hawaii	39.3	7.1
Idaho	55.8	15.4
Illinois	71.6	25.6
Indiana	62.2	8.5
Iowa	79.8	12.1
Kansas	66.4	8.6
Kentucky	47.1	7.4
Louisiana	36.9	7.4
Maine	57.8	17.9
Maryland	70.3	21.9
Massachusetts	87.2	11.8
Michigan	72.6	1.2
Minnesota	89.5	18.2
Mississippi	47.7	8.1
Missouri	76.4	21.5
Montana	77.3	4.5
Nebraska	55.6	11.1
Nevada	37.9	1.7
New Hampshire	80.6	9.7
New Jersey	65.0	36.0
New Mexico	40.7	5.6
New York	67.6	19.7
North Carolina	72.4	16.4
North Dakota	40.0	6.0
Ohio	63.7	17.9
Oklahoma	57.1	4.1
Oregon	76.9	16.9
Pennsylvania	61.5	13.1
Rhode Island	46.7	13.3
South Carolina	55.8	9.3
South Dakota	65.6	26.6
Tennessee	53.7	8.8
Texas	54.1	6.0
Utah	70.5	41.0
Vermont	62.6	11.8
Virginia	58.8	15.4
Washington	66.4	15.9
West Virginia	49.2	7.7
Wisconsin	82.0	32.0
Wyoming	51.5	24.2

^a^Hospitals reporting the provision of virtual visits in the American Hospital Association Annual Survey (AHAAS) or functioning tele-capacity in the AHAAS Information Technology Supplement were identified as providing some form of telehealth and therefore tele-capacity. All other hospitals were recorded as not providing telehealth.

^b^ICU: intensive care unit.

## Discussion

As the number of COVID-19 cases increases, the US population’s interest in telehealth also increases. However, the level of population interest did not correlate with the proportion of hospitals providing telehealth services in the United States. These observations may raise a question of whether hospitals and health care systems have the capacity to meet the increasing health care demand in their service area. Although telehealth can help to improve the triage and coordination of care for patients with COVID-19 [3-5], telecommunication infrastructures in US hospitals may lack the capability to address the ongoing health care needs of patients with other health conditions. There is still ongoing debate regarding the quality of care delivered using telehealth. Future studies should explore how the expansion of telehealth services influences the providers’ scope of practice (eg, chronic condition management and surveillance, other preventive care services) and patient outcomes (eg, quality of care, patient experience, and unintended outcomes).

This study is limited by the use of internet search data to assess population interest, which may not reflect genuine population interest. However, the utility of Google Trends and its representativeness of US population has been demonstrated [9,12]. Our study is also limited by our measures of telecapacity, which were limited in at least 2 ways: (1) We were limited to hospitals that responded to the AHAAS. Although a majority of hospitals responded and the AHAAS is commonly used for research purposes in the literature, the missing responses limit the generalizability of our findings to only hospitals responding to the AHAAS. (2) Our definitions of telecapacity were limited to hospitals, and the provision of nonhospital teleservices were not identified. Although there are nonhospital providers of telemedicine services, the need for tele-ICU services is expected to be more relevant in the hospital setting. Thus, our inclusion of the tele-ICU measure demonstrates, to some degree, the provision of teleservices geographically, which are relevant to current and forthcoming patient needs related to COVID-19.

Our findings have important implications for the nation’s current effort to address COVID-19. The CMS’ rapid response under the Coronavirus Aid, Relief, and Economic Security (CARES) Act is expected to help hospitals and other health care facilities manage their capacity and workflow [2,13]. Subsequently, increased use of telehealth services may help flatten the transmission curve overall [3,4]. However, hospitals in some regions may not have the capacity to handle the surge in telehealth and remote critical patient care. Moreover, there is uncertainty about whether hospitals can actively expand their telehealth platforms or implement a new system if they have not adopted them previously because the CMS’ waiver only extends until the end of the COVID-19 emergency. Additional investment is needed, at least in regions with low telehealth adoption, to increase capacity for population demand and empower hospitals with the flexibility to plan patient care transition against the impending patient surge [3,14]. For those who were not using telehealth to optimum capacity, structured guidelines may be needed to stimulate the effective implementation of telehealth services [15,16]. Health care decision makers may also need to appreciate the potential role of tele-ICU that enables remote ICU care by connecting intensivists or critical care teams to hospitals with limited capacity [7]. Expanding tele-ICU capability could be a promising strategy throughout this pandemic, given the shortfalls of ICU beds in rural hospitals and the growing number of patients in need of intensive care [3,17].

## Abbreviations

AHAAS: American Hospital Association Annual Survey
CARES: Coronavirus Aid, Relief, and Economic Security Act
CMS: Centers for Medicare and Medicaid Services
COVID-19: coronavirus disease
IT: information technology
RSV: relative search volume
Tele-ICU: telemedicine intensive care unit
